# Unusual DNA packaging characteristics in endoreduplicated *Caenorhabditis elegans* oocytes defined by *in vivo* accessibility to an endogenous nuclease activity

**DOI:** 10.1186/1756-8935-6-37

**Published:** 2013-11-01

**Authors:** Sam Guoping Gu, Barbara Goszczynski, James D McGhee, Andrew Z Fire

**Affiliations:** 1 Department of Pathology, Stanford University School of Medicine, 300 Pasteur Drive-L302, Stanford, CA 94305, USA; 2 Department of Genetics, Stanford University School of Medicine, Stanford, CA 94305, USA; 3 Department of Biochemistry and Molecular Biology, Alberta Children’s Hospital Research Institute, University of Calgary, Calgary AB T2N 4 N1, Canada; 4 Department of Molecular Biology and Biochemistry, Rutgers the State University of New Jersey, Piscataway, NJ 08854, USA

## Abstract

**Background:**

Germ cells in animals are highly specialized to preserve the genome. A distinct set of chromatin structures must be properly established in germ cells to maintain cell fate and genome integrity. We describe DNA-surface interactions in activated *Caenorhabditis elegans* oocytes that are revealed through the activity of an endogenous nuclease ('endocleavage’).

**Results:**

Our analysis began with an unexpected observation that a majority (>50%) of DNA from ovulated but unfertilized *C. elegans* oocytes can be recovered in fragments of approximately 500 base pairs or shorter, cleaved at regular intervals (10 to 11 nt) along the DNA helix. In some areas of the genome, DNA cleavage patterns in these endoreduplicated oocytes appear consistent from cell-to-cell, indicating coherent rotational positioning of the DNA in chromatin. Particularly striking in this analysis are arrays of sensitive sites with a periodicity of approximately 10 bp that persist for several hundred base pairs of genomic DNA, longer than a single nucleosome core. Genomic regions with a strong bias toward a 10-nt periodic occurrence of A(n)/T(n) (so-called PATC regions) appear to exhibit a high degree of rotational constraint in endocleavage phasing, with a strong tendency for the periodic A(n)/T(n) sites to remain on the face of the helix protected from nuclease digestion.

**Conclusion:**

The present analysis provides evidence for an unusual structure in *C. elegans* oocytes in which genomic DNA and associated protein structures are coherently linked.

## Background

Germ cells in animals are highly specialized to preserve the genome. A distinct set of chromatin structures must be properly established in germ cells to maintain cell fate and genome integrity [1,2]. With the goal of understanding such structures in *Caenorhabditis elegans*, a number of groups have been applying combinations of genomics, biochemistry, and genetics (reviewed in [1]).

*C. elegans* oocytes arrest at the diakinesis stage of meiotic prophase I. Oocyte chromosomes at this stage are highly condensed, giving rise to the characteristic appearance of six discrete bivalents [3]. Oocyte meiotic maturation, defined by the transition between diakinesis and metaphase of meiosis I, is triggered by a signal involving the major sperm protein (MSP) released from the sperm [4-6]. A mature oocyte signals the ovulation process by regulating the gonadal sheath cell contraction and inducing dilation of the hermaphrodite spermatheca [5], and then becomes fertilized as it passes through the spermatheca. In the absence of sperm (for example*,* in mutant *C. elegans* that are fully feminized), oocytes usually arrest at the diakinesis stage. However, in certain mutant strains that produce defective sperm, oocytes continuously mature and ovulate, endoreduplicating their DNA and resulting in a large number of unfertilized polyploid oocytes accumulating in the uterus. In this study, we use an endogenous nuclease activity present in these oocytes to identify an unusual chromatin structure.

## Results

### Fragmented chromatin in activated *fer-1(b232) C. elegans* oocytes

**Figure 1:**
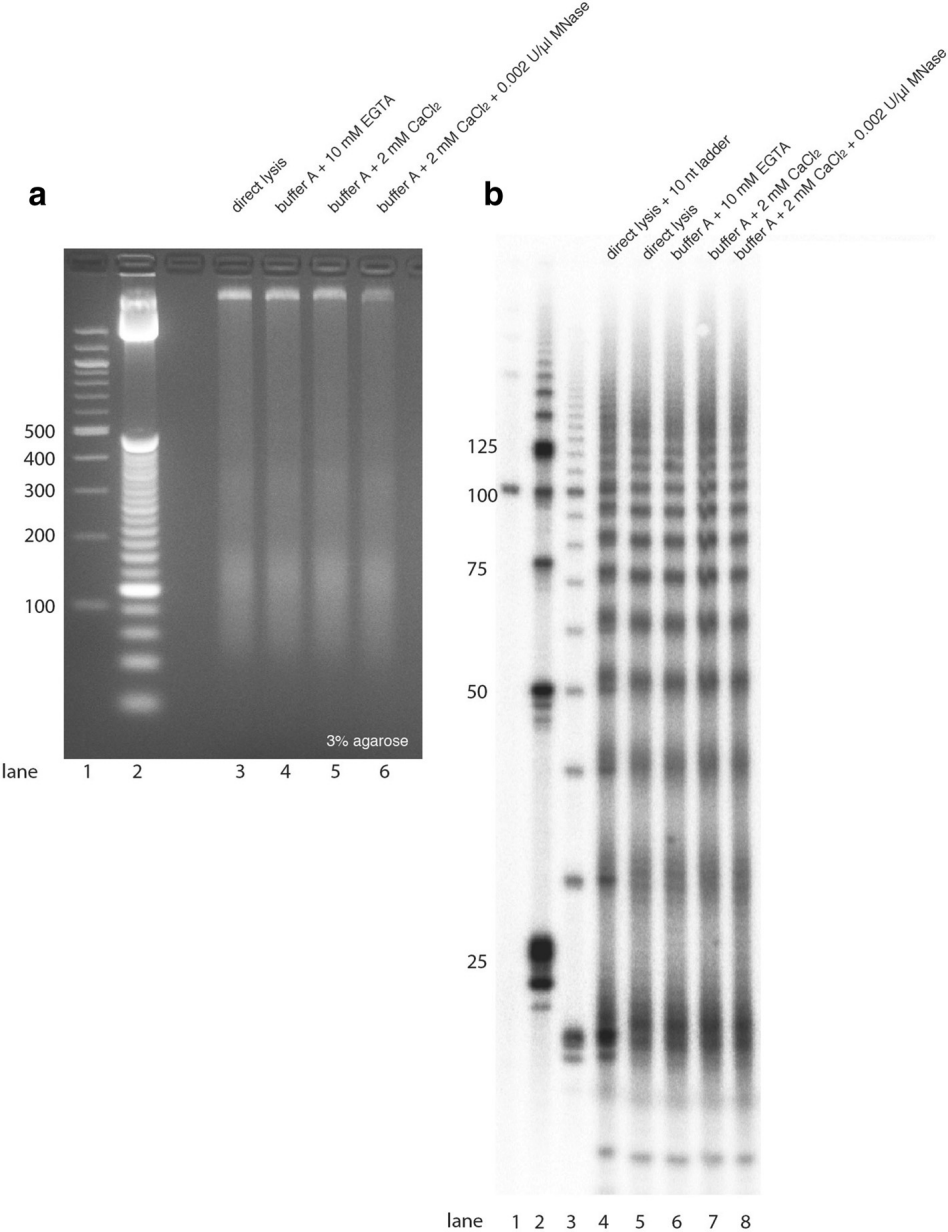
**Fragmented chromatin in activated *fer****-****1(b232) *mutant oocytes.** Genomic DNA extracted from *fer-1(b232)* oocytes was resolved on a 3% (native) agarose gel **(a)** or a denaturing 12% polyacrylamide gel (8 M urea) **(b)**. For 'direct lysis’, frozen oocytes were ground in liquid nitrogen and directly mixed with worm lysis buffer without initial thawing (see Methods). For 'buffer A + 10 mM EGTA’, 'buffer A + 2 mM CaCl_2_’, or 'buffer A + 2 mM CaCl_2_ + 0.002 U/ul MNase’, ground oocytes were mixed with corresponding buffer, followed by incubation at 37°C for 2 minutes and DNA extraction. DNA samples in panel b were radioactively labeled at the 5′ end. For 'direct lysis + 10 nt ladder’, a mixture of *fer-1(b232)* oocyte DNA and 10 nt ladder was resolved in the same lane.

**Figure 2:**
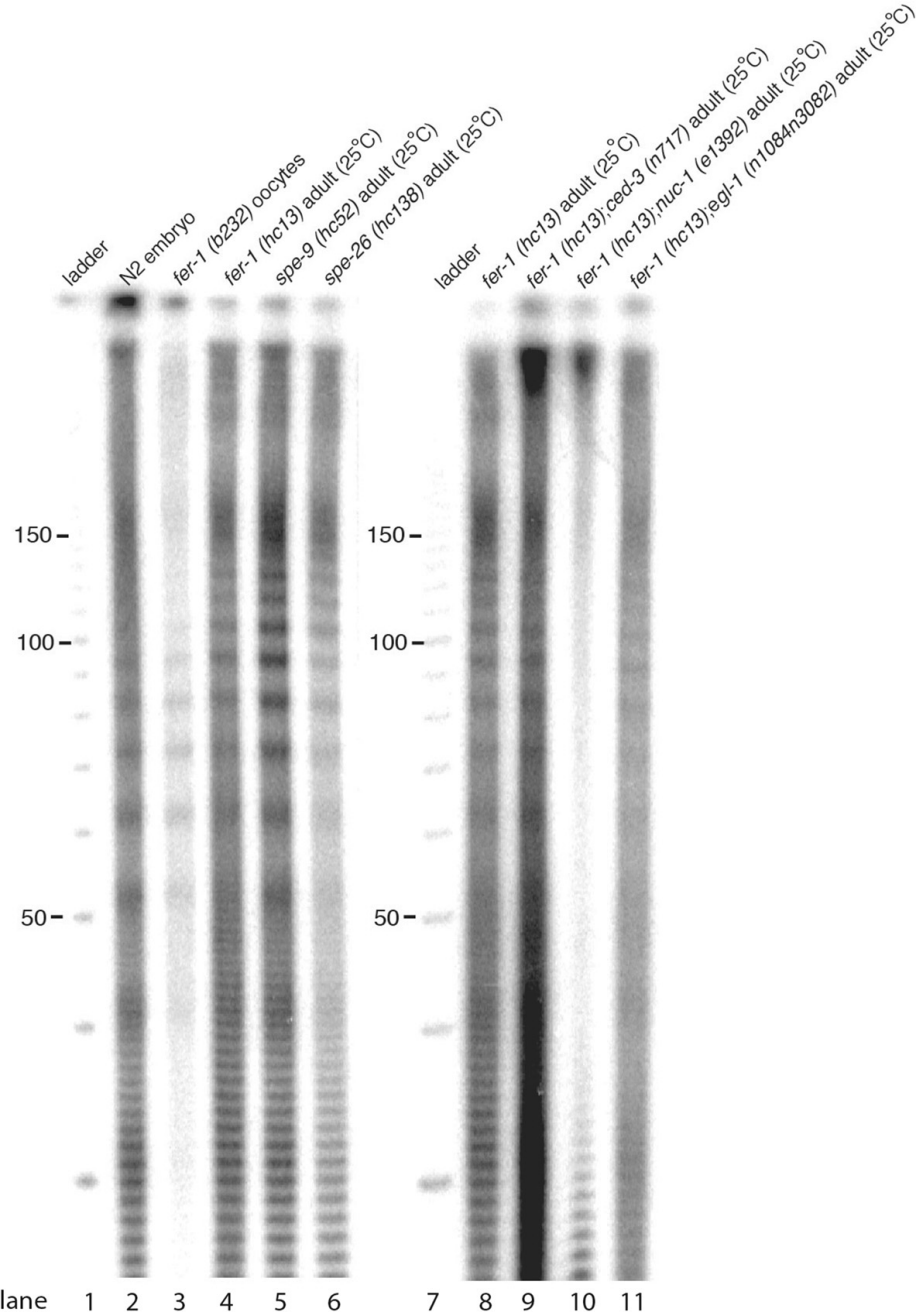
**Genetic requirement for the DNase activity present in unfertilized oocytes.** Genomic DNA extracted from purified oocytes (*'fer-1* oocytes’) or from liquid-nitrogen-flash-frozen samples (N2 (wild type) embryos and adults of various genotypes) was radioactively-labeled using T4 polynucleotide kinase and gamma-32P-ATP, and resolved on a denaturing 12% polyacrylamide gel (8 M urea). A total of 2 μg of DNA were used for labeling and PAGE analysis for all samples except DNA from isolated *fer-1(b232)* oocytes (<30 ng).

Ovulated but unfertilized oocytes have been a standard starting material for a variety of genomic and proteomic studies of *C. elegans* germline development [7-9]. These cells are a readily available germline tissue source from C. elegans, retaining transcriptional and proteomic characteristics of the oocyte lineage [8,9], although certain features (endoreduplication of DNA and accumulation over the life of the animal) distinguish them from oocytes progressing to embryogenesis in the presence of fertilizing sperm. This work began with an unexpected observation that about 50% of the DNA in these *fer-1(b232)* oocyte samples was present in fragments of <500 bp (Figure 1a). To examine the cleavage pattern in greater detail, we end-labeled DNA samples by T4 polynucleotide kinase assay and ATP (gamma-32P), resolving the products at single base resolution on denaturing 12% polyacrylamide gels (PAGEs). We observed that oocyte DNA fragments exhibit a clearly quantized size distribution with a periodicity of 10 to 11 bp on electrophoretic separation (Figure 1b). The bands define a 'ladder’ with sizes 21/22, 31/32, 41/42, 51/52 bp, *etcetera.* Such approximately 10-nt ladder patterns were not evident in undigested genomic DNA extracted from adult animals lacking activated oocytes (wild type (N2) young adult animals). Likewise the pattern was not observed in MNase-digested chromatin from L1 larvae or adult N2 animals [Additional file 1: Figure S1]. Aruscavage and colleagues [10] had observed an apoptosis-dependent population of 10 to 11 bp quantized short DNA fragments in preparations of DNA from *C. elegans* embryos, but with a considerably lower concentration relative to the total DNA present (with the low concentration of cleaved DNA in embryos likely reflecting the small fraction of cells undergoing apoptosis). With a direct comparison of the approximately 10-nt periodic ladder pattern between embryos and activated oocytes (Figure 2), we confirmed that the activated oocytes were much more strongly enriched for short quantized DNA fragments [Additional file 1: Figure S1].

### DNA fragmentation as a general property of activated *C. elegans* oocytes

To determine the generality of the observed fragmentation, we obtained unfertilized oocyte DNA from a variety of sources and by a variety of protocols. In particular, we wished to determine whether the observed approximately 10-nt ladder was dependent on either the specific genetic background of the original *fer-1(b232)* strain or the oocyte isolation procedure used. An approximately 10-nt ladder pattern similar to that observed from purified *fer-1(b232)* was observed for DNA extracted from whole animals from a different *fer-1* mutant stock (*fer-1*(*hc13*)) raised at the restrictive temperature of 25°C (Figure 2). This isolation used flash frozen animals extracted directly for DNA (without an oocyte preparation step), indicating that DNA cleavage is not due to the oocyte preparation procedure. Two other sperm-defective mutants, *spe-9*(*hc52*) [11,12] (required for sperm-oocyte interaction) and *spe-26*(*hc138*) [13] (required for spermatid formation) were tested and showed the same approximately 10-nt ladder pattern as *fer-1* mutant oocytes (Figure 2). These results are consistent with endogenous DNase activity and consequent DNA cleavage in an approximately 10-nt ladder pattern as a general property of activated *C. elegans* oocytes.

### A role for the type II DNase NUC-1 in activated oocyte endocleavage, independent of the apoptosis machinery

Chromosome fragmentation is a hallmark of apoptotic cells in mammals, as well as in lower metazoans (reviewed in [14]). Aruscavage and colleagues had showed that the endogenous cleavage activity observed in *C. elegans* embryos depends on programmed cell death machinery (the caspase-homolog CED-3) and an associated type-II DNase NUC-1 [10,15-17].

To investigate whether the canonical programmed cell death pathway and/or NUC-1 are required for the endo-cleavage activity in activated oocytes, we constructed double mutants *fer-1*(*hc13*)*;ced-3*(*n717*) and *fer-1*(*hc13*)*;nuc-1*(*e1392*), growing each at the restrictive temperature (25°C) to allow oocyte accumulation. DNA from *fer-1(hc13);ced-3(n717)* double mutant animals yields an approximately 10-base periodic ladder similar to that observed in apoptosis-competent *fer*- and *spe*-strains, indicating that CED-3 activity (and thus the canonical cell death pathway) is not required for the observed oocyte endocleavage. In contrast, the *fer-1(hc13);nuc-1(e1392)* double mutant fails to yield an evident 10-nt ladder pattern (Figure 2). These results implicate NUC-1, induced by a CED-3-independent mechanism, in the observed activated oocyte endocleavage.

### Construction of DNA libraries from endocleavage products

To understand the relation between fragmentation activities and the arrangement of DNA packaged in oocyte chromatin, we set out to characterize the precise sites of DNA cleavage. Fragmented *fer-1(b232)* oocyte DNA from different size ranges (20 to 80, 40 to 130, 80 to 230, and 130 to 430 bp) was isolated by using an agarose gel. The majority of these DNA fragments were double-stranded with a 2 to 3 nt overhang at the 3′ ends (see later section and Methods). Size-selected DNA fragments were treated with T4 DNA polymerase to produce blunt ends, followed by linker ligation. The resulting libraries were analyzed by paired-end sequencing (Illumina HiSeq 2000 sequencing system), allowing both ends to be mapped. With the criterion that paired reads match perfectly to a unique position of the reference genome, approximately 6.1, 8.0, 15.6, and 15.5 million alignments were obtained by this process for the 20 to 80, 40 to 130, 80 to 230, and 130 to 430 nt size ranges, respectively. We also performed single-end Illumina sequencing for endocleaved oocyte DNA in the 40 to 90, 90 to 140, and 140 to 240 nt size ranges.

The size of captured DNA fragments can be assessed by the distance between paired reads. The distributions of the distances between paired reads show approximately a 10-bp periodicity (Additional file 1: Figure S2), consistent with the DNA electrophoresis profile on the denaturing PAGE (Figure 1b). The peak sizes determined from sequencing are approximately 2 to 3 nt shorter than the sizes determined from a denaturing gel (Additional file 1: Figure S2 and Figure 1b), a difference that can be explained by a 2 to 3 nt single stranded overhang at the 3′ end on each strand (See Methods section and Additional file 1: Figure S3). The 130 to 430 nt size range DNA had a more continuous distribution, with maximal frequency at 145 nt. We note, however, that an approximately 10-bp periodic component remains in the 130 to 430 nt size range (for example*,* peaks at 178, 189, 199 nt) [Additional file 1: Figure S2].

### Activated oocyte DNA is constrained in its rotational positioning at the genomic level

The detailed structure of chromatin in a region can determine which face of the DNA double helix is protected by an underlying surface (for example*,* core histone octamer) and which face is accessible. At any given locus in the genome, the accessible face can be random among individual cells (that is*,* variable rotational positioning) or, in the other extreme, a particular face can be consistently accessible in all cells (that is, exhibiting a constrained rotational positioning). The 10-nt ladder appearance of the fragmented DNA in activated *fer-1(b232)* oocytes indicates that the endogenous DNA cleavage events tend to occur periodically for every helical turn in the DNA double helix.

**Figure 3:**
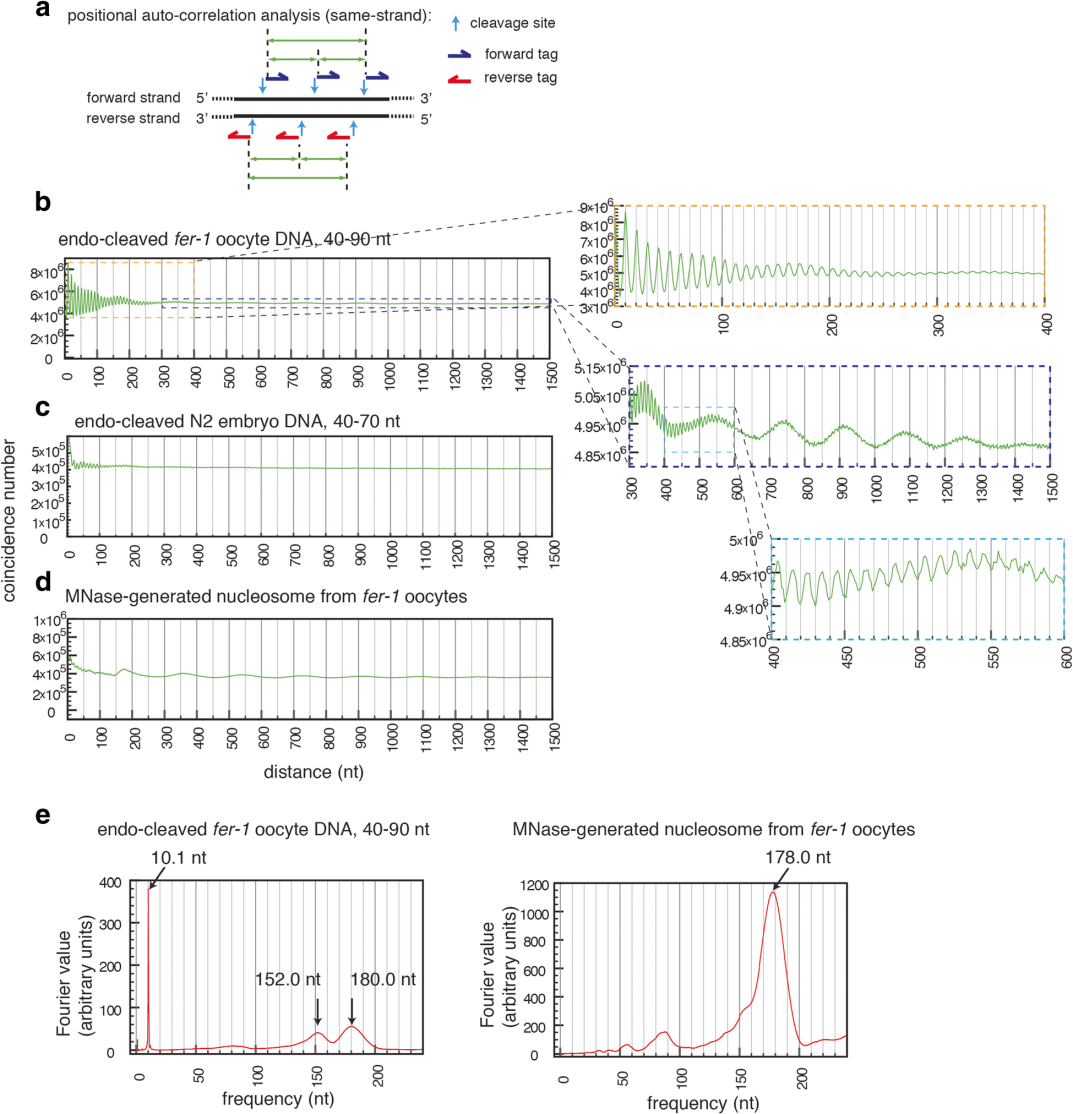
**Autocorrelation analysis of DNA fragments from *Caenorhabditis elegans fer****-****1(b232) *oocytes and wild type (N2) embryos. (a)** A schematic diagram showing an example of global positional correlation analysis. For each pair of tags that match to the same strand, the distance between the tags is recorded. **(b-d)** The coincidence of tag pairs is plotted as a function of the distance between two tags that match to the same strand for endo-cleavage DNA fragments (40 to 90 nt size range, single-end Illumina sequencing tags) from activated *fer-1(b232)* oocytes (b), 40 to 70 nt fragments from N2 embryo (c), or MNase-generated nucleosome core DNA from activated *fer-1(b232)* oocytes (d). **(e)** Fast Fourier transform analysis of coincidence over positional correlation analysis.

Positional auto-correlation analysis is a sensitive method to detect non-random DNA packaging configurations (for example, local positional constraints or long-range phasing characteristics) [18-20]. The algorithm is briefly described here. For each cleavage site (site A), the distance to each of the nearby cleavage sites (site B) is recorded. In this analysis, tags associated with sites A or B are mapped to the same strand of genomic DNA. To avoid the same pair being counted twice, site B is set downstream of site A (when referenced to the plus strand of genome) (Figure 3a). The number of such coincidences is then plotted as a function of the distance between site A and B. As an explicit example, if there are 2 and 3 tags that are mapped to sites A and B, respectively, and A and B are separated by a distance of 20 bp in the genome, the y-axis is incremented by 6 (2 times 3) at the distance of 20 bp (x-axis). If rotational positioning were completely flexible, the coincidence number would tend to be a flat function of distance between sites A and B. If rotational positioning were to be constrained, the coincidence numbers should reveal a periodic signal that corresponds to the DNA helical period.

The auto-correlation analysis for activated oocyte DNA fragments reveals a prominent approximately 10-nt periodic signal for the coincidence numbers (for example*,* peaks at 10, 21, 31, 41, 51, 62, 72, and 82 nt, *etcetera*) (Figure 3b), indicating that the cleavage sites in the activated oocyte chromatin have a strong tendency for consistent placement on an 'accessible’ face of the DNA double helix in different cells. This type of result suggests an underlying surface that may locally protect one face of the DNA from digestion while leaving the other face open to the enzyme. A previous whole-genome analysis of DNase I-generated chromatin fragments using human cells revealed a similar 10-nt periodic signal for DNase I-sensitive sites; however the observed phasing character was restricted to a distance that would be contained in a single mononucleosome [21]. In contrast, the 10-nt periodic signal observed in the *C. elegans* oocyte endo-cleaved chromatin fragments is maintained in aggregate over a distance ranging up to 500 bases and above, indicated by the 10-nt periodicity in this region of the autocorrelation plot (Figure 3b). By this analysis, 34% of the autocorrelation signal with a 100-nt window derives from sites with constrained rotational positioning (8.9 for 101 to 200 nt, 2.8% for 201 to 301 nt, 1.1% for 301 to 400 nt, and 0.64% for 401 to 500 nt). Fast Fourier transform (FFT) analysis of this signal indicated that the periodicity of the coincidence frequency is 10.1 nt (Figure 3c). However, we note that the Fourier analysis may represent a situation that in reality is considerably more complex than can be modeled with a single peak - indeed DNA in different physical and biological configurations is known to have helical periodicity ranging between 10 and 11 (for example*,*[22]) with the underlying physical situation expected to vary both between cell types and (as a consequence of DNA sequence, base composition, or underlying physical structure) between regions in the genome. Numerous large-scale chromatin structures have been proposed in diverse systems, each with different detailed consequences in terms of the balance of helical periodicity across any localized region [23-25]. Experimental analysis of accessibility [26,27], likewise supports a somewhat variable periodicity within the nucleosomal repeat that varies somewhat for different sub-nucleosomal regions (in particular around the dyad).

To obtain an indication of the extent of periodic structure underlying the DNA as a function of position in the genome, we performed the autocorrelation analysis separately for endo-cleavage ends that occur in each of six chromosomes. All chromosomes exhibit similar degrees of rotational positioning in this analysis [Additional file 1: Figure S4]. We also performed the autocorrelation analysis separately for endo-cleavage ends that occur within introns or exons. Both exonic and intronic ends exhibit similar high degrees of rotational positioning [Additional file 1: Figure S4]. These observations implicate an underlying periodic structure as a consistent and extensive feature of activated oocyte chromatin.

When the auto-correlation analysis was performed for the MNase-generated DNA fragments from the longer *fer-1(b232)* oocyte chromatin, the coincidence numbers oscillate with additional periodicity that corresponds to an approximately 178-nt nucleosome-like repeat length (that is*,* nucleosome core DNA + spacer) (Figure 3d and 3e), consistent with at least a fraction of DNA in the oocyte preparations being packaged in regularly spaced, positionally constrained nucleosomes. The prominent approximately 10-nt phasing signal observed for the endocleaved oocyte DNA fragments is absent in the MNase-generated nucleosome core DNA fragments.

When the auto-correlation analysis was performed for the endo-cleavage DNA fragments from wild type embryos, the degree of non-random rotational positioning is approximately 5-fold lower than that observed for *fer-1(b232)* oocyte endo-cleaved DNA fragments in the size range of 1 to 100 nt (and approximately 7-fold in the size range of 101 to 200 nt) (Figure 3c); the stronger amplitude and persistence of autocorrelation deriving from *fer-1(b232)* oocytes argues for differentiating features of oocyte chromatin that produce greater long-range periodicity and greater cell-to-cell rotational consistency than was observed in the somatic embryo tissue.

In summary, the prominent approximately 10-nt periodic signals in the oocyte auto-correlation analyses indicate that a particular face of the activated oocyte DNA in a large fraction of the genome is preferentially cleaved by the endogenous DNase activity. For this portion of the genome, we can infer that the activated oocyte DNA has been operationally constrained in its rotational positioning relative to an underlying protective surface.

### Packaging of activated oocyte DNA at the 5′ ends of H3K4me3-anchored genes exhibits unusual phasing characteristics

Genome-wide nucleosome mapping studies from a number of model organisms have shown nucleosome positioning that appears to be variable for a substantial fraction of the genome [18,28]. A small fraction of nucleosomes, on the other hand, are constrained to occupy specific positions. These so called positioned nucleosomes are often found near transcription start sites of active genes [20,29-32]. The first nucleosome downstream of the transcription start site (conventionally referred to as the plus-one nucleosome) often exhibits the highest degree of positional constraint. In addition, the plus-one nucleosome tends to include a distinct set of histone variants (for example*,* H3.3) [33] and post-translationally modified histones (for example*,* H3K4me2/3) [34].

**Figure 4:**
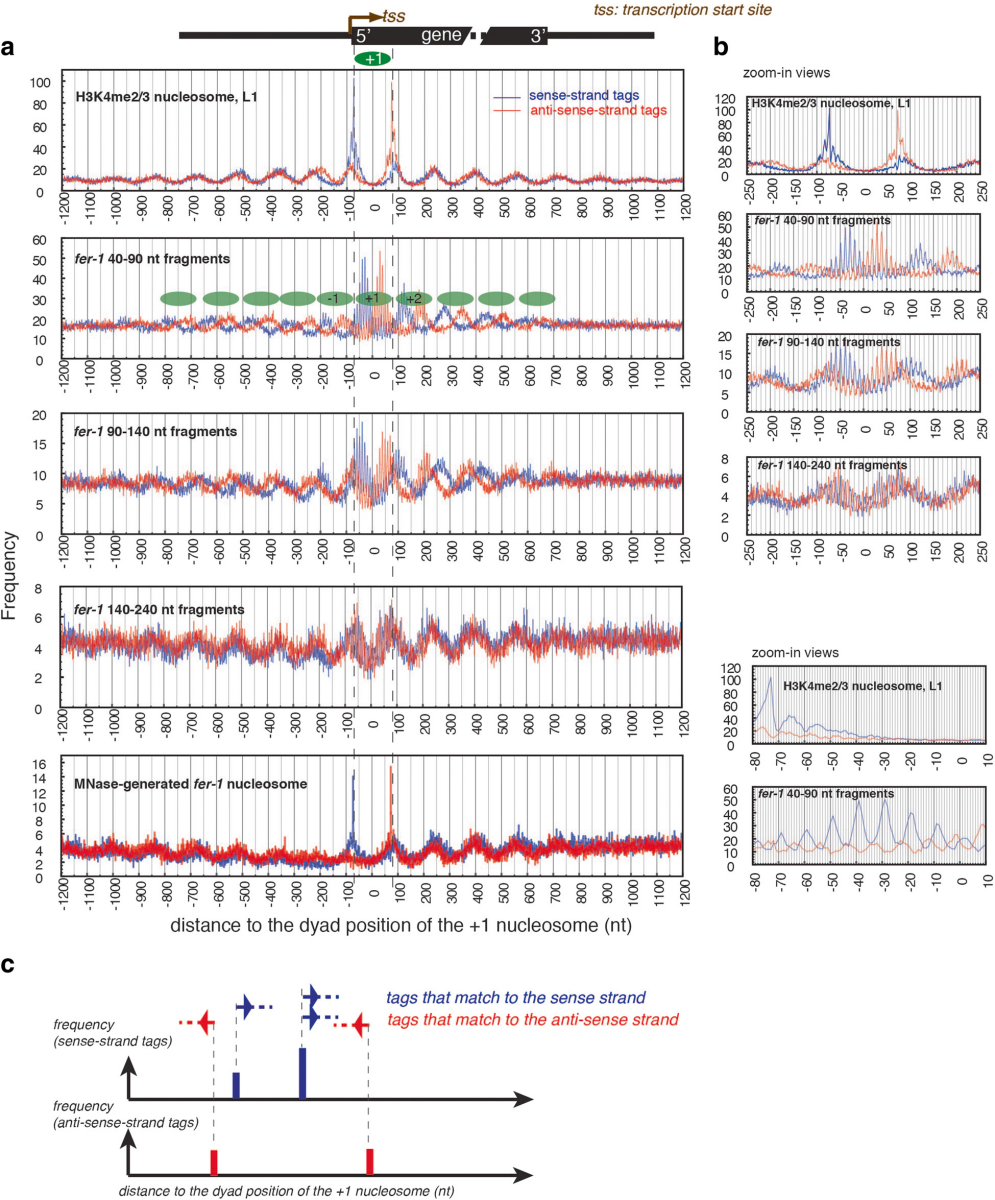
**Endo-cleavage profiles at the 5′ ends of genes for the activated oocyte chromatin. (a)** The number of tags was plotted as a function of distance to the dyad of the 'plus-one’ nucleosome, defined by peak H3K4me2/3 nucleosomes near transcription start sites for the 3903 'H3K4me2/3-anchored’ genes [20]. Tags that mapped to sense-strand and anti-sense-strand were plotted separately. A set of nucleosome cores are indicated by ovals (scaled to indicate a size of 147 nt). To contrast short and longer-scale periodicities, several expanded views are plotted in panel **(b)**. **(c)** A schematic diagram showing the analysis.

Previously, we assigned the plus-one nucleosomes for 3903 *C. elegans* genes by mapping nucleosomes that are enriched for H3K4me2/3 [20]. House-keeping genes in *C. elegans* are highly over-represented in this set of H3K4me2/3-anchored genes. To evaluate the expression status of these genes in oocytes, we used serial analysis of gene expression (SAGE) data from purified oocytes [35]. Out of the 3903 H3K4me2/3-anchored genes, at least 1,593 (40%) appear to be expressed in oocytes, as evidenced by the presence of ≥2 oocyte SAGE tags (we note that the presence of SAGE reads in oocytes does not imply oocyte-restricted expression; indeed, expression of many of these genes might be expected to be broadly expressed 'housekeeping’ genes). To characterize chromatin in active genomic regions, we examined activated oocyte DNA fragments at the 5′ ends of the 1,593 H3K4me2/3-anchored genes. In Figure 4, we plot the average frequency of the activated oocyte DNA fragment ends as a function of distance from the dyad position of the plus-one nucleosome (see Figure 4c for the scheme). Ends that match the sense strand of genes are plotted separately from ends matching the anti-sense strand. This analysis reveals two overlaying patterns: (1) a long-range oscillation that corresponds to regularly spaced nucleosomes with approximately 160-bp repeat length, and (2) a local oscillation with approximately 10-nt periodicity (Figure 4).

Two additional features of the endo-cleavage in activated oocyte chromatin were revealed upon detailed examination of the plots in Figure 4.

1. The ends of the activated oocyte DNA fragments tend to be within regions that are packaged into nucleosome cores in mixed-tissue preparations. For example, at the plus-one nucleosome position (marked by peak frequencies of MNase-generated nucleosome core DNA ends at -73 and 73 nt for the sense- and anti-sense-strand tags, respectively, Figure 4), the sense-strand tags of the activated oocyte DNA of 40 to 90 nt size range have the highest frequencies in the first half (highest frequencies occur at positions -39 and -29 nt). The profile of anti-sense-strand tags is a near mirror image of the sense-strand tags with the reference to the dyad position of the plus one nucleosome.

2. The plus-one nucleosome in activated oocytes exhibits the highest degree of constraint on the apparent rotational positioning, indicated by the most dramatic approximately 10-nt periodic oscillation of the activated oocyte DNA ends at this region. DNA in what would be the plus-two nucleosome has a strong tendency to be constrained in the same rotational positioning as the plus-one nucleosome. Remarkably, the approximately 10-nt phasing character is maintained through the dyad positions of these two nucleosome positions and even the linker region. The constrained rotational positioning shared by plus-one and plus-two nucleosome regions diminishes outside of these nucleosome regions (compared to the plus-two nucleosome, the minus-one nucleosome shows a weaker but still significant tendency to be constrained in the same rotational positioning as the plus-one nucleosome). We note the possibility that the underlying structure in these regions may be non-nucleosomal in some or all oocytes (or may be a very unusual nucleosome-like structure): whether the surface of interaction being revealed by these cleavages is indeed a pair of nucleosomes remains to be determined.

### Periodic A(n)/T(n) clusters (PATCs) characteristic of germline-expression in *C. elegans* are positioned on the inaccessible surface of activated oocyte DNA

DNA with short A(n)/T(n) sequences that are distributed along one face of the DNA double helix exhibits unusual curvature and a tendency for the A(n)/T(n) sequence to face inward towards the nucleosome, at least *in vitro*[36-40]*.* A significant fraction of the *C. elegans* genome has a strong approximately 10-nt periodic A(n)/T(n) signal [41-43], with high PATC frequency associated with oocyte-expressed genes [41]. Although this DNA can exhibit DNA bending *in vitro*[44] (and can be packaged into nucleosomes [18,20,28]) the structural consequences of this sequence characteristic for the organization of chromatin are presently unknown.

**Figure 5:**
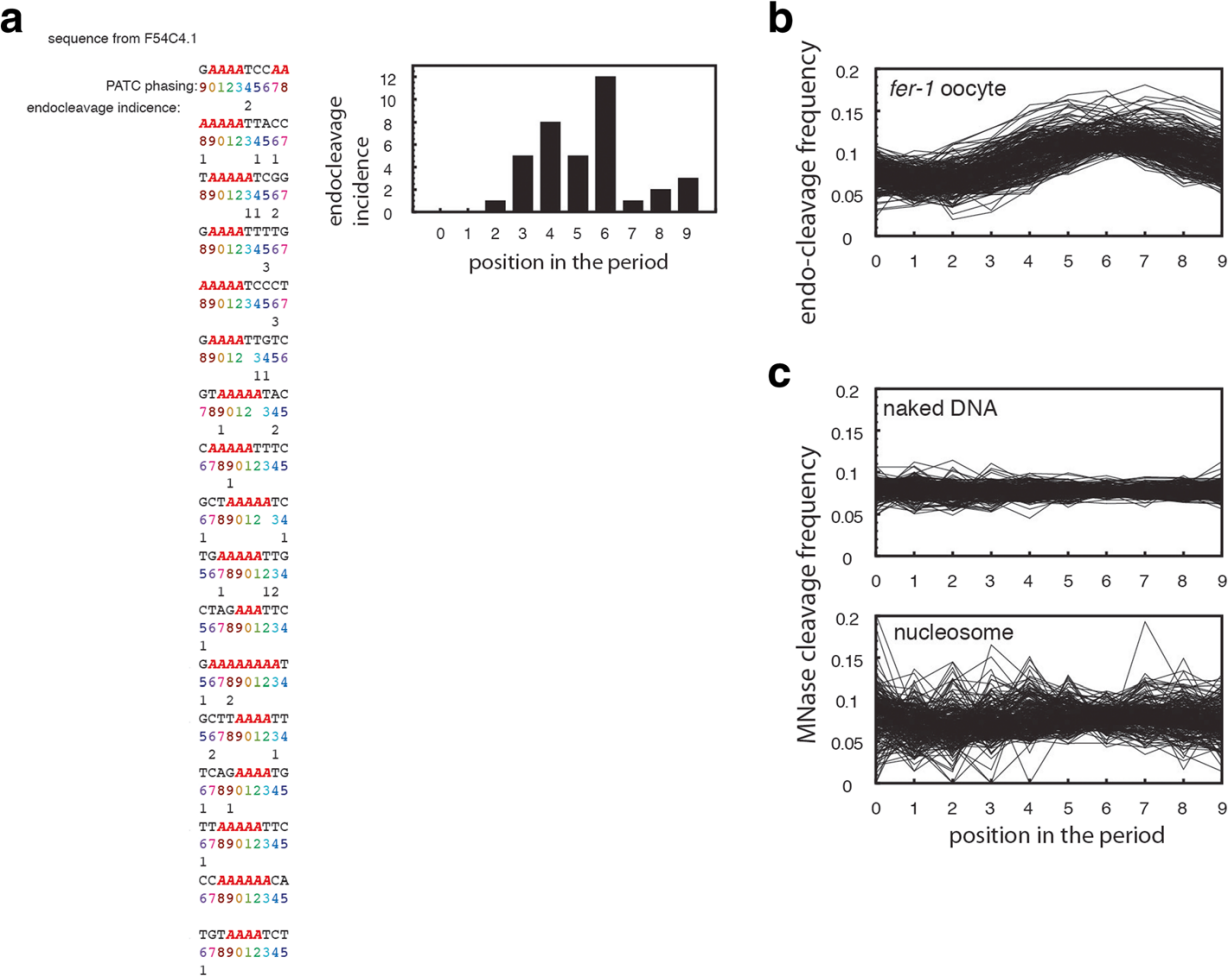
**Rotational positioning of the activated oocyte chromatin in regions that are enriched for the periodic A(n)/T(n) clusters (PATC). (a)** An example of examining the endo-cleavage events for different rotational phasing of the DNA double helix. A DNA segment of strong PATC character was selected from the intronic sequence of gene F54C4.1. Integer numbers from 0 to 9 below each line of sequences are used to indicate the PATC phasing number (that is*,* the positions relative to the A(n)/T(n) face, which is designated as 0). In a second line, the numbers of endo-cleavage events are indicated. The accessibility for each of the ten phase bins is measured by summing the endo-cleavage events occurring at the same face. The bar graph plots the endo-cleavage frequency as a function of the PATC phasing number. **(b)** For genome-wide analyses, the total endo-cleavage sites for each of the ten phase bins were separated into 256 classes according to the cleavage site sequence (NN|NN). The comparison of DNA accessibility among the ten phase bins was then performed by using cleavage sites of the same sequence, generating a total of 256 comparisons. For each of the 256 tetramer sequences, cleavage frequency (normalized by the frequency of the corresponding tetramer at the cleavage sites) is plotted as a function of the PATC phasing number for activated *fer-1(b232)* oocytes. **(c)** Same analysis performed on MNase-digested naked DNA and nucleosome cores.

To test if the 10-nt periodic A(n)/T(n) signal is associated with the rotational positioning in activated oocytes, each position in the PATC regions was assigned an integer phase (0 to 9] based on the DNA sequence alone, indicating a position relative to the A(n)/T(n) face (a schematic example is shown in Figure 5a). The accessibility for each of the ten bins was measured, summing the respective cleavage incidences. To avoid any confounding effect caused by intrinsic DNA sequence bias of the endogenous cleavage activity, the cleavage events at different PATC positions (0 to 9) were evaluated for each of the 256 classes based on the bases flanking potential cleavage sites (N_-2_N_-1_|N_1_N_2_). Regardless of the sequence composition at the cleavage site, we observed a consistent bias for the A(n)/T(n)-rich face to be protected from cleavage in the activated oocyte DNA (Figure 5b). Conversely, the face of the DNA helix that is, opposite to the A(n)/T(n) sites consistently shows the highest accessibility (face 6 Figure 5b). This pattern is not observed for MNase digested nucleosome core DNA (Figure 5c).

## Discussions and conclusions

The patterns of DNA fragmentation in activated *C. elegans* oocytes provide evidence for a large-scale chromatin organization in which long segments of DNA (>500 bp) are consistently organized on a surface that constrains accessibility of one helical face. That these organized segments are larger than individual nucleosomes argues either (i) for a stereotyped multi-nucleosome structure that might allow an uninterrupted approximately 10-bp periodicity, (ii) for a larger 'mega’ nucleosome-like structure that might accommodate several hundred base pairs of DNA, or (iii) for a large non-nucleosomal surface that might organize DNA. We consider each of the three models to be potentially valid hypotheses for further study.

A number of previous structural discussions (see for example, [24,45,46] for relevant discussion) have dealt with questions related to the potential persistence of an approximately 10-bp periodicity in sequence accessibility over multiple adjacent conventional nucleosomes. While nucleosomes separated by a variable spacer length would be expected to lose helically periodic accessibility at separations significantly beyond a single unit nucleosome length, certain fixed or constrained linker lengths would allow retention of a periodic pattern. Such arrangements might have the effect (with potential advantage to the organism) of allowing a single underlying periodicity in some regions of the genome to constrain incremental sliding of nucleosomes in response to lateral forces, while potentially increasing nucleosome dissociation in response to such forces.

While conventional single-octamer nucleosome-based structures are certainly prevalent in virtually every system analyzed, there have been additional observations (see [23] for review) suggesting flexibility in the underlying structure that might be expected under specific constraints to also allow larger histone-based complexes ('Mega-nucleosomes’?) as scaffolds for larger segments of DNA. While certainly requiring confirmation and further analysis, such larger structures are consistent with early studies on at least one system with actively replicating DNA [47].

Beyond the category of nucleosome-like protein: DNA structures, additional non-nucleosomal surfaces within the nucleus (and the semi-consistent association of these surfaces with the DNA) could account for a periodicity as we have observed; candidate surfaces might include nuclear lamina and envelope structures, meiotic condensation cores, and (potentially) yet-to-be-discovered protein-DNA interfaces.

Whatever their structural basis, the biochemical patterns revealed by our analysis match features associated with promoter organization and periodic nucleotide sequence composition in germline-expressed *C. elegans*[41] genes, suggesting (despite the endoreduplicated character of oocytes isolated in bulk from *C. elegans*) that the chromosome organization described here would have been present and functionally relevant on a sufficient evolutionary timescale to influence the underlying sequence, either through selection at the organismal level or through mutational biases introduced by the anisotropic activity.

## Methods

### DNA extraction

*C. elegans* oocytes were purified from the *fer-1(b232)* mutant worms grown at the restrictive temperature of 25-26°C as described [35]. Oocytes were flash frozen upon harvest. Frozen oocytes were ground to a fine powder in liquid nitrogen. For direct lysis (that is, no micrococcal nuclease treatment), DNA was isolated by incubating the worm sample with 400 μl lysis buffer (100 mM Tris-Cl, pH 7.5, 0.1 M NaCl, 1% SDS, 50 mM EDTA, 0.2 mg/ml protease K) at 65°C for 1 hour, followed by extraction with an equal volume of phenol/chloroform (1:1), extraction with an equal volume of chloroform, and by precipitation with ethanol (1 ml). Approximately 5 to 20 μg of DNA was obtained from this procedure. In cases where micrococcal nuclease (MNase) treatment was applied, liquid-nitrogen-ground oocytes (or similar samples from other stages) were directly resuspended in buffer A (15 mM Hepes-Na, pH 7.5, 60 mM KCl, 15 mM NaCl, 0.15 mM beta-mercaptoethanol, 0.15 mM spermine, 0.15 mM spermidine, 0.34 M sucrose) containing 0.5 mM PMSF, 1/100 dilution of HALT protease and phosphatase inhibitor cocktail (Thermo Scientific), 1 mM DTT, and 2 mM CaCl_2_. For micrococcal nuclease (MNase) digestion, 0.1 ml oocyte crude lysate was first pre-warmed to 37°C for one minute, then treated with 0.002, 0.02, or 0.1 U/l MNase (Roche). The reaction mix was incubated at 37°C for two minutes and digestion was then stopped by adding EGTA to a final concentration of 20 mM. DNA was isolated by incubating the worm sample with 400 μl lysis buffer (100 mM Tris-Cl, pH 7.5, 0.1 M NaCl, 1% SDS, 50 mM EDTA, 0.2 mg/ml protease K) at 65°C for 1 hour, followed by organic extraction and DNA precipitation as above.

### DNA library preparation

DNA was treated with 0.5 U/μl T4 polynucleotide kinase (NEB) in 1x T4 DNA ligase buffer (NEB, containing 1 mM ATP) at 37°C for 1 hour to phosphorylate 5′ ends and dephosphorylate 3′ ends. Ends were then blunted in a reaction containing 0.75 U/μl of T4 DNA polymerase (NEB), dNTP (100 μM each, Roche), and 1x NEB buffer 1 at 12°C for 15 min. DNA was then ligated to previously annealed DNA oligonucleotides AF-SG-133 (5′ Pi-AGATCGGAAGAGCTCGTATGCCGTCTTCTGCTTG-OH 3′) and AF-SG-134 (5′ OH-CCCTACACGACGCTCTTCCGATCT-OH 3′) (NEB Quick Ligation Kit). DNA captured by linker oligonucleotides was resolved using 6% polyacrylamide (acrylamide:bis-acrylamide = 19:1) gels containing 8 M urea, followed by PCR amplification with primers AF-SG-135 (5′ OH-AATGATACGGCGACCACCGAGATCTACACTCTTTCCCTACACGACGCTCTTCCGATCT-OH 3′) and AF-SG-137 (5′ OH-CAAGCAGAAGACGGCATACGAGCT-OH 3′). We carefully selected cycle numbers for which product levels have not saturated (that is, product levels were still able to increase substantially with additional cycles); this protocol ensures that the majority of amplified segments are still annealed to a true complement, avoiding reannealing distortion in the resulting sequence libraries [48]. After separating PCR products on 2% agarose gels, DNA bands of the expected size were extracted (Qiagen Gel Extraction Kit (Invitrogen); omitting the 50°C heating step), followed by massively parallel DNA sequencing using Illumina HiSeq 2000 sequencing system.

### Accession numbers

All sequencing data used in this study have been deposited in GEO ( GSE46499).

### Characterization of the ends of fragmented DNA from activated oocytes

We radioactively labeled 5′-ends of *fer-1(b232)* oocyte DNA by using T4 polynucleotide kinase and ATP gamma [32] P, followed by treatment with T4 DNA polymerase and dNTPs to produce blunt ends. End-labeling and T4 DNA polymerase reactions were performed when DNA was double-stranded. T4 DNA polymerase converts 5′ or 3′ single-stranded overhangs to blunt ends by either DNA polymerase or 3′-5′ exonuclease activity, respectively. When the T4 DNA polymerase-treated oocyte DNA was resolved on a denaturing PAGE, we observed that the sizes of oocyte DNA fragments consistently decreased by 2 to 3 nt when compared with the untreated sample [Additional file 1: Figure S3], indicating a 2 to 3-nt single-stranded overhang in the 3′ ends of oocyte DNA fragments (*that is,* two nearby cleavage events on the opposite strands are staggered by 2 to 3 nt).

SAGE data were obtained from the Genome BC *C. elegans* Gene Expression Consortium http://elegans.bcgsc.bc.ca/.

## Abbreviations

PATCs: periodic A(n)/T(n) clusters; SAGE: serial analysis of gene expression.

## Competing interests

The authors declare that they have no competing interests.

## Authors’ contributions

Experiments were conceived in discussions among all authors. *fer-1* Oocytes were prepared by B. G. and J.D.M. Experiments and data analyses for Figure 1–5 were performed by S.G.G. and A.F. Overall discussion of the data and implications involved all authors, and the manuscript was written by S.G.G., J.D.M. and A.F. All authors read and approved the final manuscript.

## Supplementary Material

Additional file 1 **Figure S1.** DNA extracted from MNase digested chromatin and naked DNA treated with. MNase digestion was resolved on a 3% agarose gel (**a**) or a denaturing 12% polyacrylamide gel (8 M urea) (**b**).**Figure S2.** Size distributions of fragmented *fer-1(b232)* oocyte DNA determined by paired-end Illumina sequencing for each of the four libraries prepared from fragmented DNA in different size ranges (20–80, 40–130, 80–230, and 130–430 bp). **Figure S3.** Characterization of the ends of fragmented *fer-1(b232)* oocyte DNA. Denaturing PAGE analysis of fragmented *fer-1(b232)* oocyte DNA treated with T4 DNA polymerase and a full set of dNTP. **Figure S4.** A positional correlation analysis of endo-cleaved DNA fragments from *fer-1(b232) C. elegans* oocyte for each chromosome (**A**) A positional correlation analysis of endo-cleaved DNA fragments from *fer-1(b232) C. elegans* oocyte (**B**) and wild type (N2) embryo (**C**) chromatin using endo-cleavage ends that fall in intronic or exonic sequence. **Figure S5.** (**A**) Coverage by endo-cleaved DNA fragments (left panels) or MNasegenerated nucleosome DNA (right panels) from *fer-1(b232) C. elegans* oocytes was plotted as a function of position for each of the *C. elegans* six chromosomes. (**B**) A density scatter plot comparing the coverage of endo-cleaved DNA fragments and coverage of MNase-generated nucleosome DNA from *fer-1(b232) C. elegans* oocyte for each one-kb window throughout the genome. The density of dots are indicated by color. (**C**) Aggregate coverages of endo-cleaved DNA fragments or MNase-generated nucleosome DNA from *fer-1(b232) C. elegans* oocyte were plotted as a function of positions at the 5′ end and 3′ ends of annotated *C. elegans* genes. Genes are grouped as high, median, and low based on the levels of oocyte expression. Click here for file
