# Supplementary material for: Unusual DNA packaging characteristics in endoreduplicated Caenorhabditis elegans oocytes defined by in vivo accessibility to an endogenous nuclease activity
Source: Epigenetics Chromatin. 2013 Nov 1;6:37. doi: 10.1186/1756-8935-6-37 (PMC3819648; doi:10.1186/1756-8935-6-37)
Supplement: Additional file 1 — Figure S1. DNA extracted from MNase digested chromatin and naked DNA treated with. MNase digestion was resolved on a 3% agarose gel (a) or a denaturing 12% polyacrylamide gel (8 M urea) (b).Figure S2. Size distributions of fragmented fer-1(b232) oocyte DNA determined by paired-end Illumina sequencing for each of the four libraries prepared from fragmented DNA in different size ranges (20–80, 40–130, 80–230, and 130–430 bp). Figure S3. Characterization of the ends of fragmented fer-1(b232) oocyte DNA. Denaturing PAGE analysis of fragmented fer-1(b232) oocyte DNA treated with T4 DNA polymerase and a full set of dNTP. Figure S4. A positional correlation analysis of endo-cleaved DNA fragments from fer-1(b232) C. elegans oocyte for each chromosome (A) A positional correlation analysis of endo-cleaved DNA fragments from fer-1(b232) C. elegans oocyte (B) and wild type (N2) embryo (C) chromatin using endo-cleavage ends that fall in intronic or exonic sequence. Figure S5. (A) Coverage by endo-cleaved DNA fragments (left panels) or MNasegenerated nucleosome DNA (right panels) from fer-1(b232) C. elegans oocytes was plotted as a function of position for each of the C. elegans six chromosomes. (B) A density scatter plot comparing the coverage of endo-cleaved DNA fragments and coverage of MNase-generated nucleosome DNA from fer-1(b232) C. elegans oocyte for each one-kb window throughout the genome. The density of dots are indicated by color. (C) Aggregate coverages of endo-cleaved DNA fragments or MNase-generated nucleosome DNA from fer-1(b232) C. elegans oocyte were plotted as a function of positions at the 5′ end and 3′ ends of annotated C. elegans genes. Genes are grouped as high, median, and low based on the levels of oocyte expression. [file 1756-8935-6-37-S1.pdf]

Supplemental figures

Figure S1: DNA extracted from MNase digested chromatin and naked DNA treated with MNase digestion was resolved on a 3% agarose gel (**a**) or a denaturing 12% polyacrylamide gel (8M urea) (**b**).

Figure S2: Size distributions of fragmented *fer-1(b232)* oocyte DNA determined by paired-end Illumina sequencing for each of the four libraries prepared from fragmented DNA in different size ranges (20-80, 40-130, 80-230, and 130-430 bp).

Figure S3: Characterization of the ends of fragmented *fer-1(b232)* oocyte DNA. Denaturing PAGE analysis of fragmented *fer-1(b232)* oocyte DNA treated with T4 DNA polymerase and a full set of dNTP.

Figure S4: A positional correlation analysis of endo-cleaved DNA fragments from *fer-1(b232) C. elegans* oocyte for each chromosome (A). A positional correlation analysis of endo-cleaved DNA fragments from *fer-1(b232) C. elegans* oocyte (B) and wild type (N2) embryo (C) chromatin using endo-cleavage ends that fall in intronic or exonic sequence.

Figure S5: (A) Coverage by endo-cleaved DNA fragments (left panels) or MNase-generated nucleosome DNA (right panels) from *fer-1(b232) C. elegans* oocytes was plotted as a function of position for each of the *C. elegans* six chromosomes. (B) A density scatter plot comparing the coverage of endo-cleaved DNA fragments and

coverage of MNase-generated nucleosome DNA from *fer-1(b232) C. elegans* oocyte for each one-kb window throughout the genome. The density of dots are indicated by color. (C) Aggregate coverages of endo-cleaved DNA fragments or MNase-generated nucleosome DNA from *fer-1(b232) C. elegans* oocyte were plotted as a function of positions at the 5' end and 3' ends of annotated *C. elegans* genes. Genes are grouped as high, median, and low based on the levels of oocyte expression.

Figure S1

a

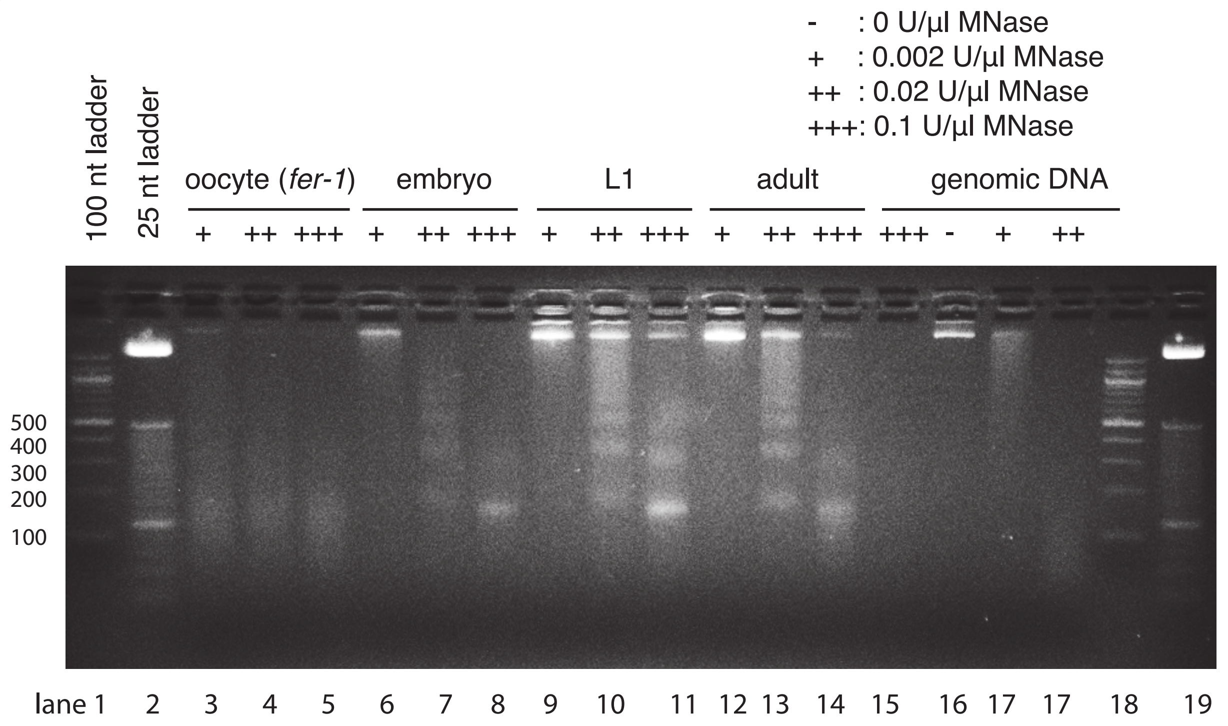

- : 0 U/µl MNase
+ : 0.002 U/µl MNase
++ : 0.02 U/µl MNase
+++: 0.1 U/µl MNase
100 nt ladder
25 nt ladder
oocyte (*fer-1*)
embryo
L1
adult
genomic DNA
+ ++ +++ + ++ +++ + ++ +++ + ++ +++ +++ - + ++
500
400
300
200
100
lane 1 2 3 4 5 6 7 8 9 10 11 12 13 14 15 16 17 17 18 19

b

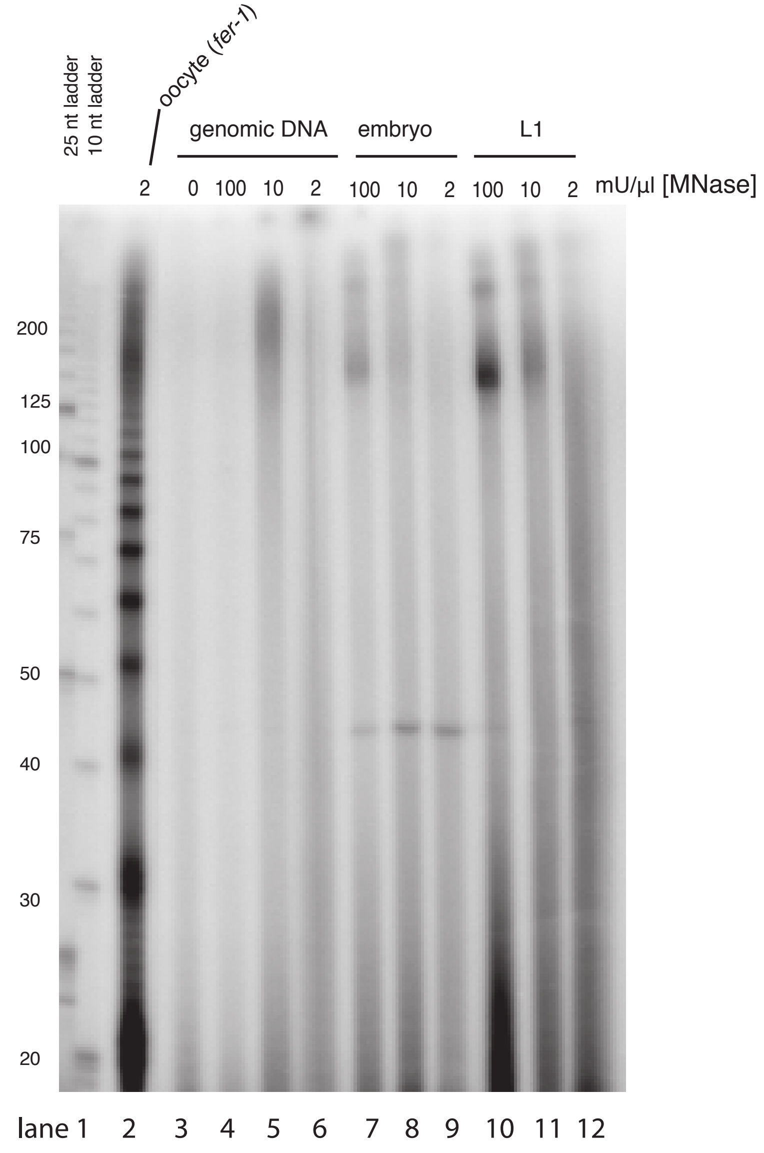

25 nt ladder
10 nt ladder
oocyte (*fer-1*)
genomic DNA
embryo
L1
2 0 100 10 2 100 10 2 100 10 2 mU/µl [MNase]
200
125
100
75
50
40
30
20
lane 1 2 3 4 5 6 7 8 9 10 11 12

Figure S2

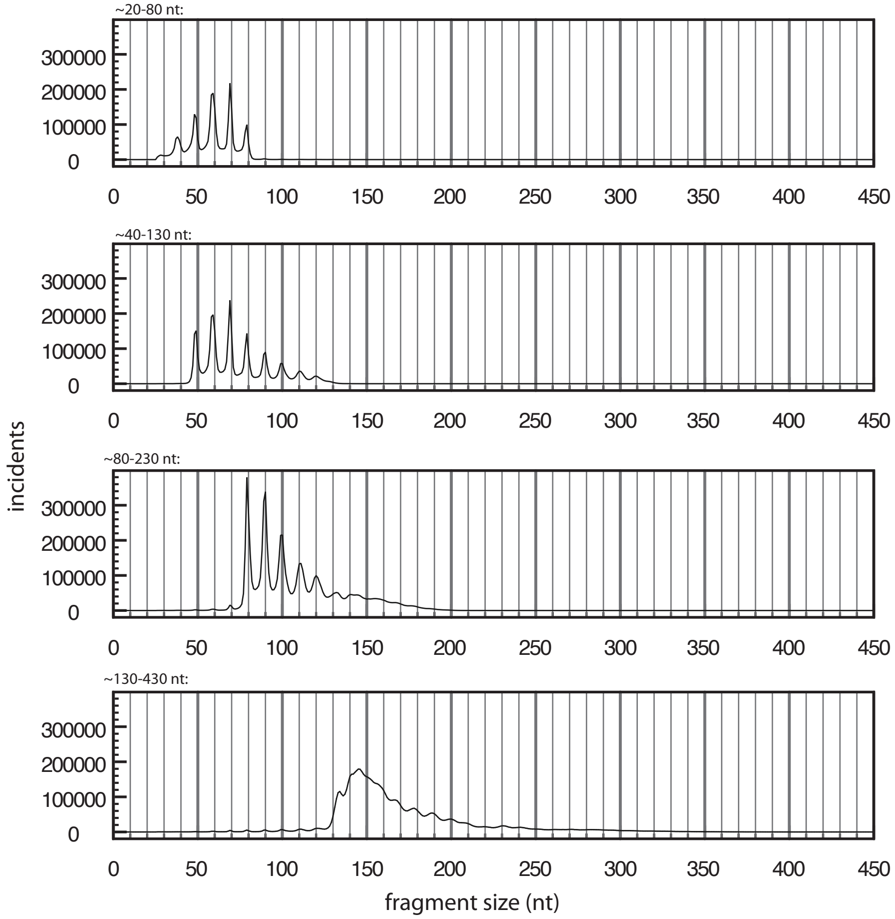

~20-80 nt:
~40-130 nt:
~80-230 nt:
~130-430 nt:
300000
200000
100000
0
0
50
100
150
200
250
300
350
400
450
incidents
fragment size (nt)

Figure S3

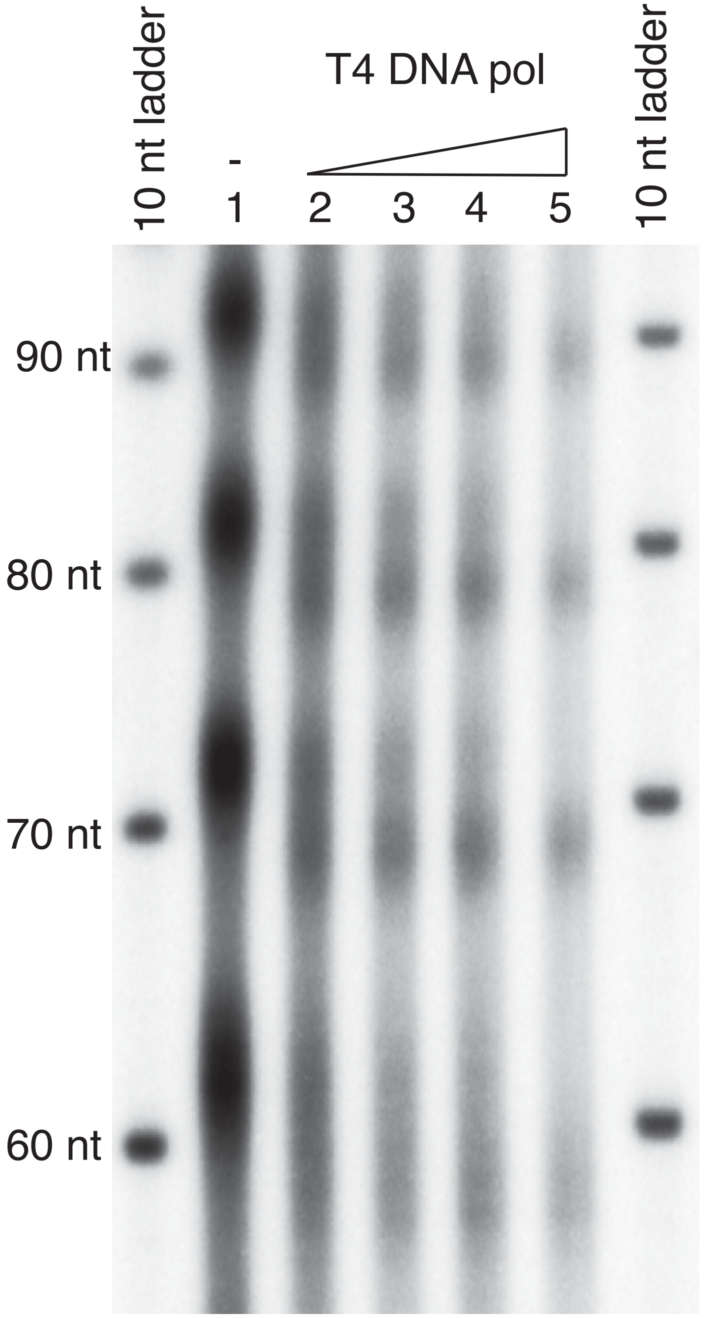

Figure S4

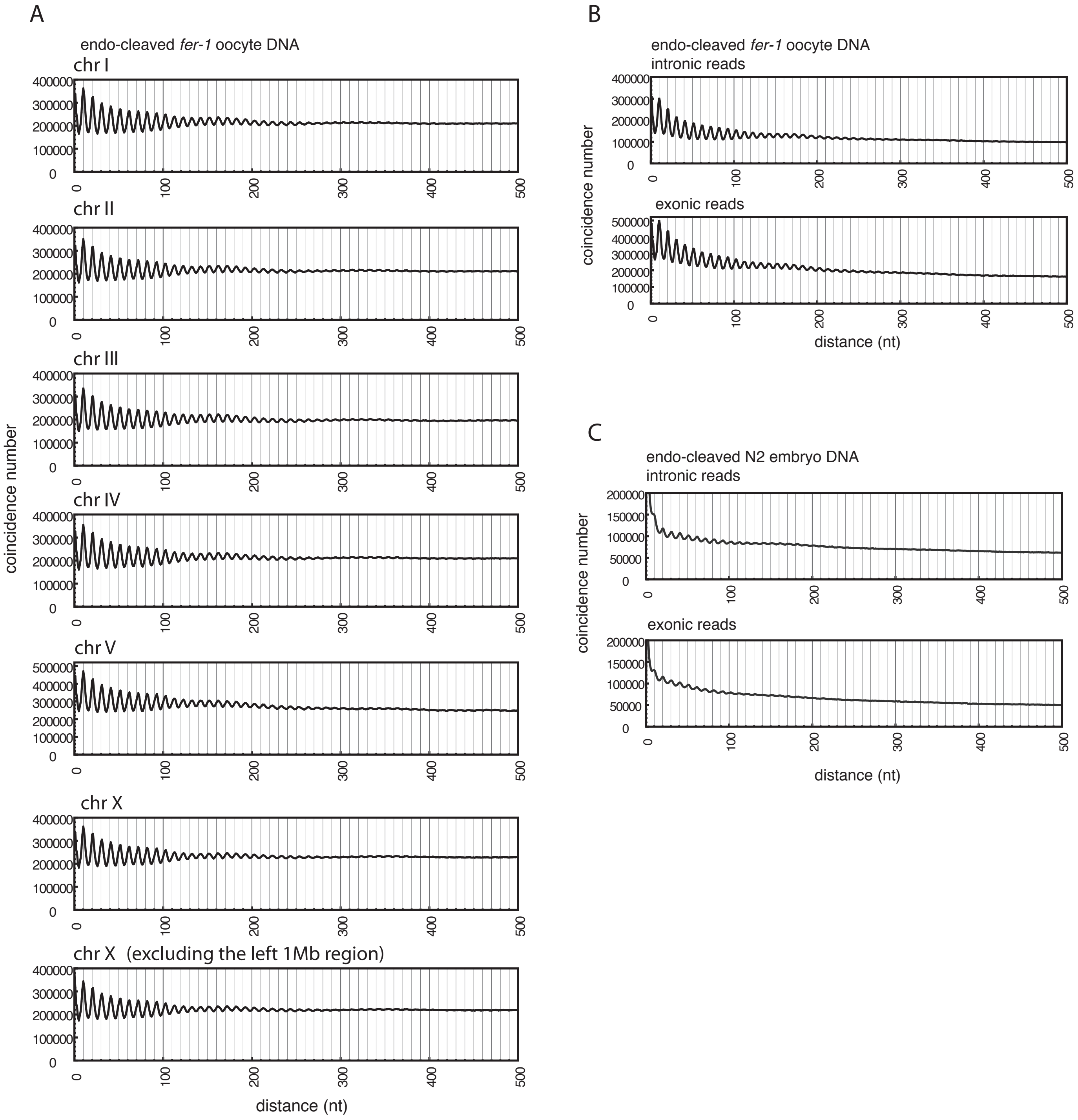

A
endo-cleaved *fer-1* oocyte DNA
chr I
chr II
chr III
chr IV
chr V
chr X
chr X (excluding the left 1Mb region)
coincidence number
distance (nt)
B
endo-cleaved *fer-1* oocyte DNA
intronic reads
exonic reads
coincidence number
distance (nt)
C
endo-cleaved N2 embryo DNA
intronic reads
exonic reads
coincidence number
distance (nt)

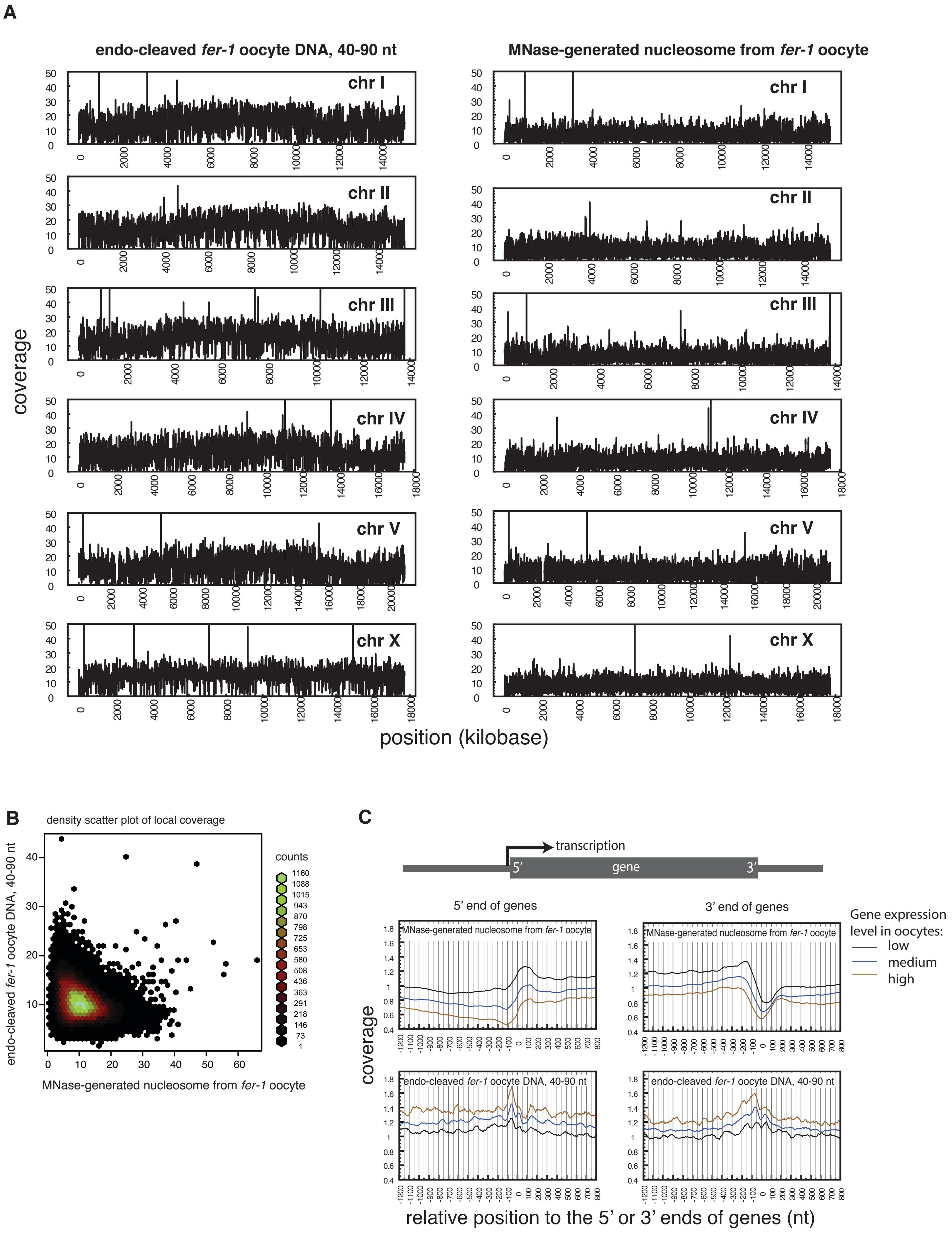
A
endo-cleaved fer-1 oocyte DNA, 40-90 nt
MNase-generated nucleosome from fer-1 oocyte
chr I
chr II
chr III
chr IV
chr V
chr X
coverage
position (kilobase)
B
density scatter plot of local coverage
endo-cleaved fer-1 oocyte DNA, 40-90 nt
MNase-generated nucleosome from fer-1 oocyte
counts
1160
1088
1015
943
870
798
725
653
580
508
436
363
291
218
146
73
1
C
transcription
5′
gene
3′
5′ end of genes
3′ end of genes
Gene expression
level in oocytes:
low
medium
high
MNase-generated nucleosome from fer-1 oocyte
endo-cleaved fer-1 oocyte DNA, 40-90 nt
coverage
relative position to the 5′ or 3′ ends of genes (nt)
